# Quantitative background parenchymal enhancement and fibro-glandular density at breast MRI: Association with BRCA status

**DOI:** 10.1007/s00330-023-09592-2

**Published:** 2023-04-05

**Authors:** Rosie Goodburn, Evanthia Kousi, Clarrissa Sanders, Alison Macdonald, Erica Scurr, Catey Bunce, Komel Khabra, Mamatha Reddy, Louise Wilkinson, Elizabeth O’Flynn, Steven Allen, Maria Angélica Schmidt

**Affiliations:** 1 CRUK Cancer Imaging Centre, The Institute of Cancer Research and Royal Marsden Foundation Trust, London, UK; 2 The Royal Marsden NHS Foundation Trust, Sutton, UK; 3 St Georges University Hospitals NHS Foundation Trust, London, UK

**Keywords:** Female, Breast density, Parenchymal tissue, Magnetic resonance imaging, Biomarkers

## Abstract

**Objectives:**

To investigate whether MRI-based measurements of fibro-glandular tissue volume, breast density (MRBD), and background parenchymal enhancement (BPE) could be used to stratify two cohorts of healthy women: BRCA carriers and women at population risk of breast cancer.

**Methods:**

Pre-menopausal women aged 40–50 years old were scanned at 3 T, employing a standard breast protocol including a DCE-MRI (35 and 30 participants in high- and low-risk groups, respectively). The dynamic range of the DCE protocol was characterised and both breasts were masked and segmented with minimal user input to produce measurements of fibro-glandular tissue volume, MRBD, and voxelwise BPE. Statistical tests were performed to determine inter- and intra-user repeatability, evaluate the symmetry between metrics derived from left and right breasts, and investigate MRBD and BPE differences between the high- and low-risk cohorts.

**Results:**

Intra- and inter-user reproducibility in estimates of fibro-glandular tissue volume, MRBD, and median BPE estimations were good, with coefficients of variation < 15%. Coefficients of variation between left and right breasts were also low (< 25%). There were no significant correlations between fibro-glandular tissue volume, MRBD, and BPE for either risk group. However, the high-risk group had higher BPE kurtosis, although linear regression analysis did not reveal significant associations between BPE kurtosis and breast cancer risk.

**Conclusions:**

This study found no significant differences or correlations in fibro-glandular tissue volume, MRBD, or BPE metrics between the two groups of women with different levels of breast cancer risk. However, the results support further investigation into the heterogeneity of parenchymal enhancement.

**Key Points:**

• *A semi-automated method enabled quantitative measurements of fibro-glandular tissue volume, breast density, and background parenchymal enhancement with minimal user intervention*.

• *Background parenchymal enhancement was quantified over the entire parenchyma, segmented in pre-contrast images, thus avoiding region selection*.

• *No significant differences and correlations in fibro-glandular tissue volume, breast density, and breast background parenchymal enhancement were found between two cohorts of women at high and low levels of breast cancer risk*.

**Supplementary Information:**

The online version contains supplementary material available at 10.1007/s00330-023-09592-2.

## Introduction

Breast cancer is the second most diagnosed cancer and the second most common cause of female cancer death in the UK, with similar incidence and mortality rates in other developed countries [1]. The main independent risk factors for breast cancer in women include age, inherited changes in BRCA1 and BRCA2 genes (i.e. BRCA carrier status), and breast density [2–6]. Breast density, or fibro-glandular density, refers to the proportion of fibro-glandular tissue in the breast relative to adipose tissue. When assessed using X-ray mammography (XRM), breast density is usually classified into one of four BI-RADS categories of breast composition [7, 8]. However, it is also possible to derive quantitative measurements using XRM [9].

MRI has several advantages over XRM for measuring breast density. MRI provides high-resolution, three-dimensional (3D) images while XRM relates to a two-dimensional (2D) projection image that may be affected by tissue overlap effects [10]. Additionally, MRI does not use ionising radiation or require breast compression and can be used to measure breast density in women of all ages. For these reasons, MRI breast density (MRBD) measurements are often considered the gold standard for describing breast density [11, 12].

In addition to measuring breast density, MRI can also be used to detect a phenomenon called background parenchymal enhancement (BPE). BPE refers to the increased signal increase of fibro-glandular tissue on MRI following contrast uptake. Some studies have suggested that BPE measurements could be used as additional biomarkers for predicting breast cancer risk [13], but the association between BPE and breast cancer risk is unclear [14–16]. The predictive value of high BPE in different populations is also uncertain, and it is not known whether BPE is significantly different in women at high risk of breast cancer compared to the general population [14, 15]. There are several confounding factors that may contribute to the conflicting results, including variations in study populations, hormonal stimulation, and the lack of standardised methods for measuring BPE [14–18].

In this study, we aim to compare semi-quantitative measurements of MRBD and voxelwise BPE metrics between two groups of radiologically normal subjects: BRCA carriers and subjects at population risk of breast cancer. To the best of our knowledge, no other studies have directly compared MRBD or BPE measures between these two groups, where subjects at population risk of breast cancer are not usually included in MRI studies. By examining potential differences between the two groups, we hope to determine whether MRBD and BPE measurements could provide useful biomarkers for cancer risk in risk stratification models to optimise population screening programs. To determine the consistency of our measurements, we plan to conduct statistical evaluations of the inter- and intra-user repeatability and agreement of metrics derived from the right and left breasts.

## Materials and methods

### Subjects

Two cohorts of women were recruited from separate sites: one group at high risk for breast cancer (August 2015–February 2019) and another group at low risk (May 2017–November 2019). The first, high-risk, group consisted of 35 women with genetically proven BRCA1 or BRCA2 mutations. The MRI scans for this group were obtained retrospectively from a high-risk screening program, with a gap of less than 6 months between XRM and MRI. The low-risk group comprised 30 women at population-level risk (i.e. no proven BRCA1/2 mutations), who were found to be radiologically normal after being referred to the Rapid Diagnostic and Assessment Centre for breast investigations. This second group of women was recruited prospectively and underwent breast MRI within 6 weeks of XRM.

Inclusion and exclusion criteria were established to control for factors that may contribute to variations in XRM breast density and BPE within the population. The primary difference between the two groups was their genetic predisposition to develop breast cancer. Inclusion criteria for both groups included the following: (i) age between 39.5 and 50.5 years, (ii) pre-menopausal status, and (iii) MRI screening within 6 weeks of XRM. Exclusion criteria included the following: (i) previous breast cancer diagnosis or treatment, (ii) treatment or medication between XRM and MRI, and (iii) hormonal treatment and bilateral salpingo-oophrectomy.

This study was approved by the National Research Ethics Committee (REC 14/LO/1908) and complies with the Declaration of Helsinki and local data protection regulations. All prospective participants provided written consent. The study has been registered in the NIH research database (NCT03684733).

### MRI examination and protocol standardisation

All MRI scans were performed on 3-T scanners (Ingenia and Achieva, Philips Healthcare) using 7-element rigid biopsy-compatible breast coils. The protocol for high-risk breast screening at site 1 complied with national guidelines [19] and was adapted for use at site 2, with minor adjustments made to account for hardware differences between the two systems (Table 1).

**Table 1:** Acquisition parameters for sites 1 and 2. The DCE protocol involves the acquisition of one pre- and six post-contrast datasets employing a 3D fat-suppressed, spoiled gradient-echo pulse sequence

	TR (ms)	TE (ms)	FA (deg)	Plane	Acq matrix	FOV (mm)	Voxel size (mm)	Receiver bandwidth (Hz/px)	Post-contrast temporal resolution (s)
Site 1									
2D T2w SE	4134	120	90	Axial	340 × 251	400 × 400	0.85 × 0.85 × 2	334	-
DCE	4.24	2.11	12	Axial	340 × 340	480 × 480	0.71 × 0.71 × 2	541	59.1
Site 2									
2D T2w SE	4615	120	90	Axial	340 × 251	432 × 432	0.78 × 0.78 × 2	334	-
DCE	4.24	2.11	12	Axial	340 × 340	432 × 432	0.78 × 0.78 × 2	541	59.1

*Abbreviations: 2D*, 2-dimensional; *3D*, 3-dimensional; *T2w*, T2-weighted; *SE*, spin echo; *DCE*, dynamic contrast enhanced; *TR*, repetition time; *TE*, echo time; *FA*, flip angle; *Acq*, acquisition; *FOV*, field-of-view

Although the protocols were closely aligned, possible sources of bias were investigated. BPE is known to depend on pulse sequence parameters which are often overlooked [20]. In this study, the dynamic range of the DCE-MRI sequence was investigated in both scanners to ensure correct BPE estimations (Supplementary Material). Minor differences in spatial resolution have been discussed elsewhere and are not expected to be problematic [21].

### Image processing

Image processing was performed with in-house software developed in MATLAB (Mathworks). Fibro-glandular volume, MRBD, and BPE measurements were optimised separately.

#### Whole-breast masking

To generate left and right breast masks, we applied a semi-automated, 3D region-growing algorithm [22] to non-fat-suppressed (FS), T2-weighted images using our in-house method [23]. The masks were then eroded to remove the skin and chest wall.

#### Fibro-glandular volume and MRBD estimation

**Fig. 1:**
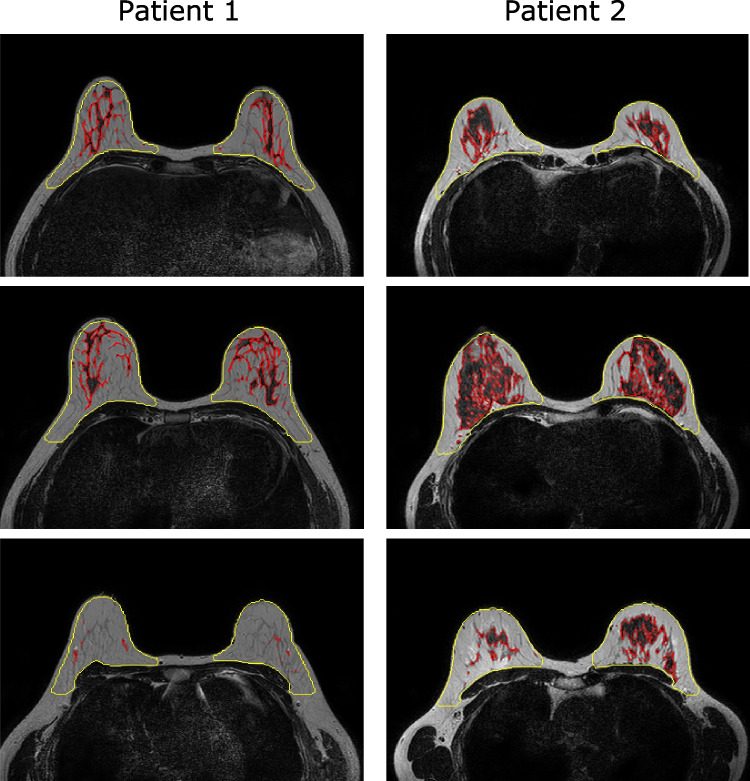
Semi-automatically generated breast masks (yellow) and parenchyma segmentations (red) overlayed on three representative axial slices of T2-weighted breast scans from two example patients. Patients were scanned at site 1 (left column) and site 2 (right column). Inferior slice locations are on the top row, while superior locations are on the bottom row. Images are displayed according to radiological convention (right breast is on left side of images)

Breast tissue was segmented on non-FS T2-weighted images (Fig. 1) to exclude bias caused by areas of fat suppression failure. Following bias-field correction with N4ITK [24], fuzzy c-means clustering [25] was used to classify each voxel as either non-fibro-glandular (e.g. adipose tissue) or fibro-glandular (parenchymal tissue). To build the segmentations, seven intensity-based clusters [26] were generated independently within each breast volume and typically assigned fibro-glandular tissue to the three clusters associated with the lowest pixel values in non-FS images. However, in cases where breast density was very low or very high, we fine-tuned the number of clusters by selecting which clusters to include as fibro-glandular tissue. This method was designed to be as objective and operator independent as practicable.

Fibro-glandular volume was calculated as the number of fibro-glandular voxels multiplied by the voxel size. MRBD was quantified as the percentage of the breast volume occupied by fibro-glandular tissue.

#### BPE estimation

**Fig. 2:**
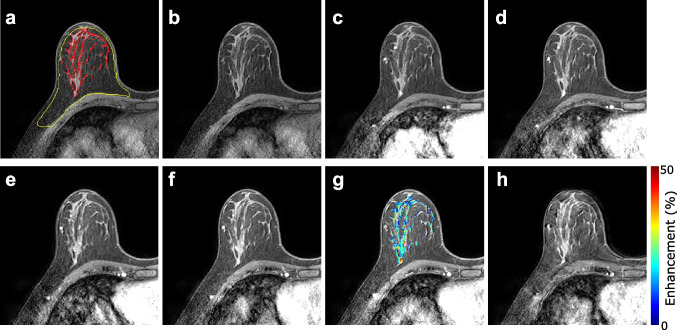
Segmentations generated from T2-weighted data were applied to DCE-MRI data (**a**) across all time points. The sequence of DCE-MRI images taken before (**b**) and after (**c–h**) bolus injection of the contrast agent is shown for an example patient. An enhancement map is shown on the 5th post-contrast image (**g**), which was the image volume with the highest median pixel value over the segmented parenchyma. Post-contrast images were acquired every 59.1 s

To estimate breast percent enhancement (BPE), we used a similar segmentation method as described above (fuzzy c-means clustering with seven clusters) to segment the fibro-glandular tissue on the pre-contrast, fat-suppressed T1-weighted images obtained from the DCE-MRI (Fig. 2). These segmentations were initialised using the whole-breast masks generated from non-FS images, resampled to match the geometry of the DCE-MRI.

By segmenting the breast parenchyma on pre-contrast images rather than post-contrast images, we aimed to probe enhancement as a distribution of values and avoid bias by not presuming enhancement, which may be position dependent. The operator intervention involved selecting which clusters to include as breast parenchyma and excluding areas of poor fat suppression that might be misinterpreted by the software. The measurement was designed to be as operator independent as possible and to include as much of the breast parenchyma as possible.

BPE maps were calculated voxelwise in segmented fibro-glandular tissue with the formula:

$$BPE\; (\mathrm{\%})=100\times {({I}_{MTP}-I}_{FTP})/({I}_{FTP})$$

where ${I}_{FTP}$ and ${I}_{MTP}$ represent pixel intensity in the first (pre-contrast) time point image (FTP) and the maximum-enhancement time point (MTP) image, respectively. The MTP image is the image in the series with the highest median pixel value over the segmented parenchymal volume.

In our analysis, we primarily considered the MTP, although data from all time points were available. The reason for this decision was that the subjects in our study were radiologically normal. In contrast, it is recommended to assess BPE in MRI of breast cancer patients at an early post-contrast time point, where cancers exhibit peak enhancement, in order to avoid including malignant tumours in the segmented fibro-glandular tissue [16]. However, this approach may result in underestimates of quantitative BPE metrics in radiologically normal women, as persistent enhancement in breast parenchyma can occur and enhancement rates may vary between subjects. Both risk groups showed maximum parenchymal enhancement at the last or second-to-last time point in the dynamic series due to the type I enhancement of the parenchyma.

Statistical BPE distribution metrics were calculated from the enhancement map across one breast fibro-glandular tissue segmentation. The median, inter-quartile range, skewness, and kurtosis of the BPE distribution were measured independently in the left and right breasts for each subject.

### Data analysis

To determine the inter- and intra-user repeatability of our method, two different software operators conducted independent measurements of fibro-glandular tissue volume, MRBD, and median BPE. Ten subjects (five from each group), with a range of breast tissue densities, were used to calculate the coefficient of variation (CV) as $(\sigma /\mu )*100\mathrm{\%}$, where $\sigma$ is the standard deviation and $\mu$ is the mean of the calculated parameter.

Bland–Altman statistics were used to explore the agreement between right and left fibro-glandular tissue volume, MRBD, and median BPE. We also considered age and weight differences between the two groups, as these factors have been associated with breast density [2].

For all estimated parameters (fibro-glandular tissue volume, MRBD, and BPE distribution metrics), histograms were plotted and assessed visually to inspect deviations from normal distribution. Differences between right and left breasts were explored accordingly using paired parametric test (paired *t*-test) and non-parametric test (Wilcoxon signed-rank test). Parametric (*t*-test) and non-parametric (Mann–Whitney *U* test) tests were also used to investigate the differences in all parameters between the high-risk and the population-risk groups. Spearman’s rank correlation coefficient, *r*, was also used to examine parameter correlations.

For all statistical comparisons, we set a significance level of *p* = 0.05 and considered *p* < 0.05 to be statistically significant. All data and statistical analyses were conducted in MATLAB.

## Results

### Subjects

Group 1 consisted of 35 women at high risk of developing breast cancer, and their MRI examinations were analysed retrospectively. For group 2, 30 women at population level of breast cancer risk were recruited prospectively, of which 26 were included in the study after four were excluded due to adverse body habitus.

Inter- and intra-user repeatability.

The intra- and inter-user CV for fibro-glandular tissue volume, MRBD, and median BPE were below 15%, as shown in Table 2.

**Table 2:** Calculated intra- and inter-user % coefficient of variation (CV) in fibro-glandular tissue volume, breast density, and median BPE

	Fibro-glandular tissue volume	Breast density	Median BPE
Intra-user %CV	8.5%	10.5%	3.0%
Inter-user %CV	13.6%	8.2%	8.4%

*Abbreviations: BPE*, breast parenchyma enhancement

### Agreement between right and left breast metrics

**Fig. 3:**
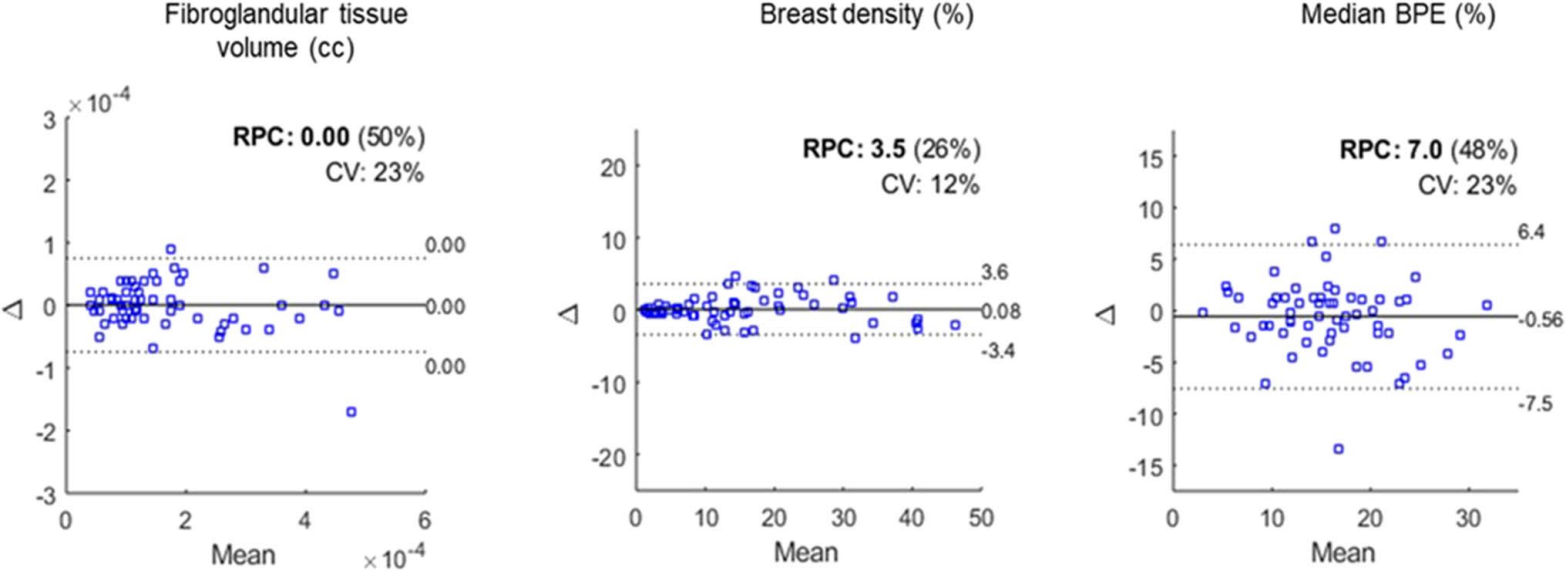
Bland–Altman plots for fibro-glandular tissue volume, breast density, and median BPE for right and left breasts. X-axis represents the mean of the right and left breast measurements and Y-axis represents the difference between the measurements from the right and left breasts. The coefficient of reproducibility, *RPC*, is defined as ± *1.96 sample standard deviation* of the differences between the paired measures (*Δ*). The solid line is the mean of differences (or the bias) and the outer dashed lines are the limits of agreement (*Δ* ± sample standard deviation of Δ * 1.96)

The coefficients of reproducibility (RPC) for fibro-glandular tissue volume, MRBD, and median BPE between the right and left breasts were found to be 0.0 cc, ± 3.5%, and ± 7.0%, respectively, when both subject groups were combined (Fig. 3). The CV for fibro-glandular tissue volume, MRBD, and median BPE between the right and left breasts were calculated as 23%, 12%, and 23%, respectively. These small mean differences (biases) between the right and left breast fibro-glandular tissue volume, MRBD, and median BPE and the corresponding levels of agreement are within clinically acceptable levels. There were no statistically significant differences between the right and left breasts for the mean values of fibro-glandular tissue, MRBD, and median BPE (*p* = 0.7, 0.9, and 0.3, respectively).

### Comparisons between high-risk and population-risk subject groups

**Fig. 4:**
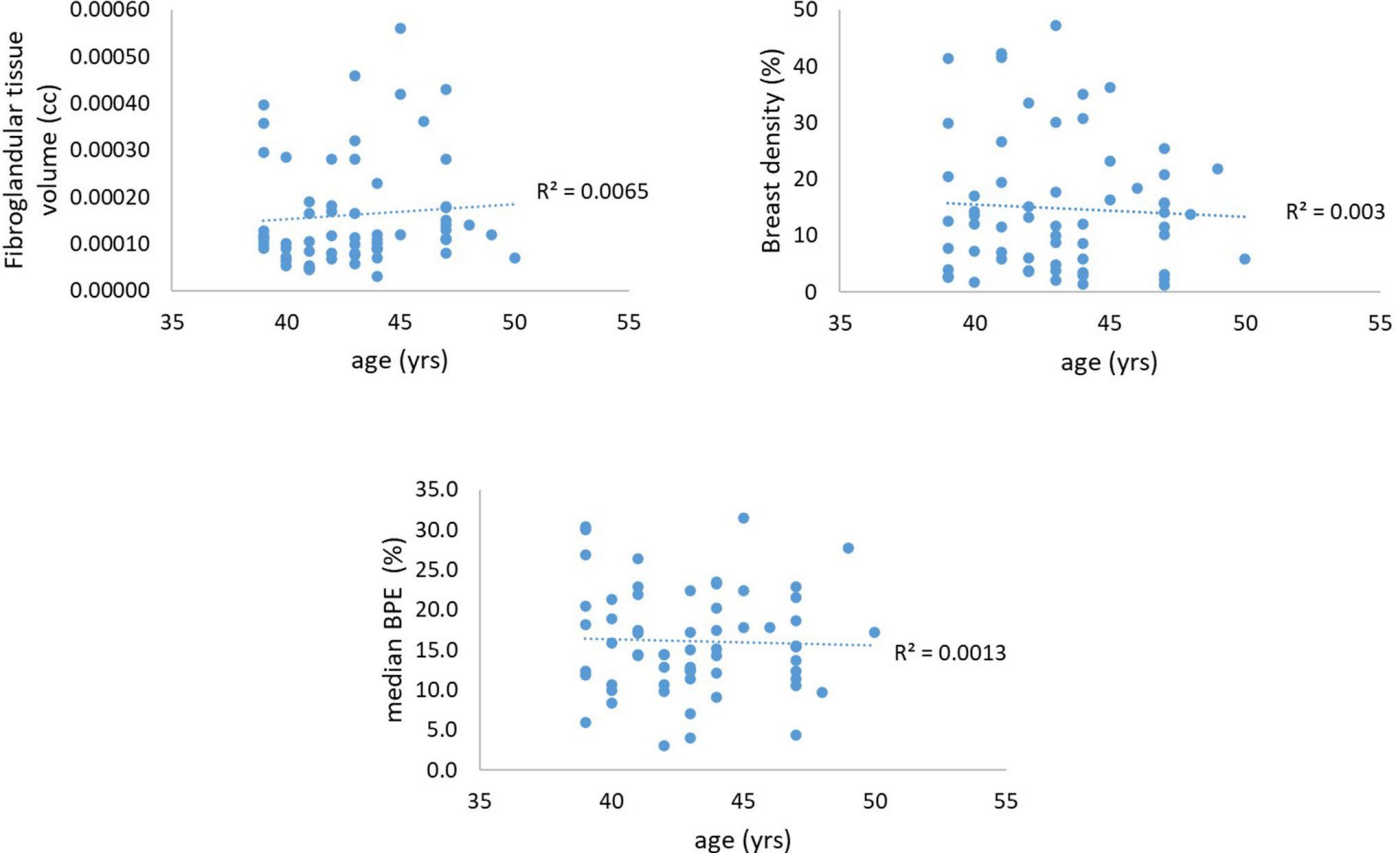
Correlation between fibro-glandular tissue volume, breast density, median BPE, and age for the entire cohort

The image quality in breast and axillae is often better on the right side, due to less residual ghosting from cardiac motion. Due to this and the agreement observed between right and left breasts, comparisons between the two groups were made using only the right breast. Although the age range for the two groups was restricted within 10 years, we found statistically significant age difference (*p* = 0.0002) between the high-risk group (41.1 ± 2.6 years) and the population-risk group (44.6 ± 2.6 years). However, there was no statistically significant difference in weight between the two groups (*p* = 0.1) (Table 3). In addition, no correlations were found between age and fibro-glandular tissue volume, MRBD, or median BPE when the two groups were considered separately (population-risk group: *r* = 0.17 (*p* = 0.4), − 0.03 (*p* = 0.9), − 0.05 (*p* = 0.8) and high-risk group: *r* =  − 0.06 (*p* = 0.7), − 0.03 (*p* = 0.9), − 0.18 (*p* = 0.3)) or together (*r* = 0.15 (*p* = 0.25), 0.002 (*p* = 1), − 0.02 (0.9), Fig. 4). The association between age and breast density is known, but the age difference between the two groups is relatively small (mean age difference = 2.7 years).

**Table 3:** Median (inter-quartile range: Q1 to Q3) for all measured parameters for high- and population-risk groups. *p*-values from parametric and non-parametric statistical tests applied to investigate differences between the two risk groups

	High-risk group	Population-risk group	Comparisons between groups *p*-values
Age (years)	42.0 (40.0 to 43.0)	44.5 (43.0 to 47.0)	0.0002 (Mann–Whitney ***U*** test)*
Weight (kg)	70.0 (60.0 to 78.0)	66.5 (59.3 to 71.8)	0.1 (Mann–Whitney *U* test)
Fibro-glandular volume (× 10^−4^ cc)	1.1 (0.8 to 1.7)	1.3 (0.9 to 2.6)	0.24 (Mann–Whitney *U* test)
Breast density (%)	12.5 (4.4 to 19.9)	11.85 (5.9 to 20.0)	0.98 (Mann–Whitney *U* test)
BPE median (%)	14.4 (11.6 to 18.5)	17.3 (12.4 to 21.4)	0.41 (Student’s *t*-test)
BPE lower quartile (%)	7.2 (4.8 to 10.8)	8.4 (5.6 to 13.2)	0.44 (Student’s *t*-test)
BPE upper quartile (%)	21.9 (18.6 to 27.9)	25.1 (21.3 to 29.3)	0.27 (Student’s *t*-test)
BPE skewness	−0.5 (− 0.6 to 0.1)	− 0.05 (− 0.6 to − 0.15)	0.08 (Student’s *t*-test)
BPE kurtosis	5.8 (4.8 to 7.7)	4.9 (3.7 to 5.7)	0.02 (Mann–Whitney ***U*** test)*

^*^ denotes statistical significance

The fibro-glandular tissue volume, MRBD, and BPE distribution metrics (median, lower quartile, upper quartile, skewness, and kurtosis) were compared between the high-risk and the population-risk groups. Only one statistically significant difference was observed: the BPE kurtosis (Table 3). For both groups, median BPE and MRBD were not significantly correlated (population-risk group: *r* = 0.4, *p* = 0.06; high-risk group: *r* = 0.18, *p* = 0.29).

Subsequently, we used linear regression analysis to investigate if the statistically significant higher BPE kurtosis for the high-risk group could be associated with age and breast cancer risk. The multivariable linear regression model showed no evidence of associations between BPE kurtosis and age, breast cancer risk, or their interaction (Table 4). Similarly, the analysis of other time points did not show any statistically significant differences in BPE between patient groups at different risk levels.

**Table 4:** Multi-variate linear regression analysis for BPE kurtosis increase with risk

Variable	Coefficient	95% confidence interval	*p*-value
Age	− 0.19	(− 0.6 to 0.2)	0.3
Breast cancer risk	− 7.05	(− 30.1 to 16.0)	0.5
Age*risk	0.19	(− 0.3 to 0.7)	0.5

*Abbreviations: BPE*, breast parenchyma enhancement

## Discussion

Two groups of radiologically normal women at different levels of risk for breast cancer were compared in terms of quantitative measures of fibro-glandular tissue volume, MRBD, and BPE. To the best of our knowledge, this is the first study of this kind. By considering radiologically normal women only, we were able to exclude changes to the breast associated with the development of cancer. This study has two notable methodological features: (i) the assessment of the dynamic range of the DCE-MRI pulse sequence used on all scanners and (ii) the segmentation of the breast parenchyma in pre-contrast images. Our segmentation methods are accessible and use data from standard clinical breast MRI exams, and we took steps to control for hormonal factors and make our quantitative measurements as operator independent as possible. To characterise the DCE-MRI pulse sequence, we used test objects to measure the level of T1 weighting, which determines the dynamic range of spoiled gradient-echo sequences used in DCE-MRI. (T1 weighting is often overlooked, even when data obtained from different equipment, field strengths, contrast agents, and protocols are combined.)

Our BPE measurement method used pre-contrast images to perform fibro-glandular tissue segmentation to include as much breast parenchyma in the evaluation as practicable. This is important because segmenting post-contrast images (or subtracted datasets) is more likely to exclude portions of the breast parenchyma where the enhancement is low or absent. We characterised the BPE pattern of each subject by a distribution of % enhancement values associated with parenchyma voxels. Our methodology was semi-automated, allowing the user to make adjustments, as validated by an intra- and inter-observer reproducibility evaluation. Although we found small differences in repeated measurements, likely due to differences in interpretation of parenchyma segmentation, the correlation between left and right measurements remained very high, indicating consistent measurements for fibro-glandular tissue volume, MRBD, and BPE.

Further validation to the design of the study is offered by both subject populations being limited to pre-menopausal women within a 10-year age range. The significant difference in age between the two groups was not expected and led us to verify that none of the measured breast attributes varied with age within the small range of ages considered, for all patient groups, as shown in Fig. 4.

No significant differences were found in fibro-glandular tissue volume, MRBD, and median BPE when the two groups of patients were compared. Our results on MRBD are consistent with those of Hu et al who did not find significant differences in fibro-glandular tissue in the breast cancer group compared with the normal control group [15]. There were, however, differences between the kurtosis of the BPE distribution in the two patient groups, although this difference can no longer be considered significant if multiple comparisons are taken into account. Multi-variate analysis demonstrated that this difference in BPE kurtosis cannot be attributed to the difference in ages in the two groups. Nevertheless, this study draws attention to the non-uniform nature of the breast enhancement, and further investigations may reveal different enhancement patterns in different patient groups. Our findings suggest that some patients may have highly vascularised sub-volumes where the enhancement is greatest. This is supported by the follow-up analysis of Dontchos et al who found larger two-dimensional areas of BPE and higher BPE signal intensity in a cohort of women who eventually developed breast cancer [27].

Previously published studies have investigated BPE as a prognostic factor of breast cancer risk. Two large recent meta-analyses found associations between increased BPE with a higher risk of breast cancer [14, 15]. Similarly, in their review study, Liao et al conclude that higher levels of BPE may reflect increased breast cancer future manifestation suggesting that BPE is an independent marker of breast cancer risk [16]. Bauer et al state that evidence supports BPE as a “probable risk factor” but further validation is required with large-scale studies, standardised protocols, and methods of analysis [28].

We believe our study contributed towards the standardisation of protocols for BPE measurements by considering the dynamic range of the sequences employed. Clinically, BPE is mainly estimated qualitatively in the four broad categories (minimal, mild, moderate, marked); however, the estimations are subjective and suffer from large inter-reader variability [16]. Where qualitative measurements are employed, it is best practice to standardise the windowing levels for image viewing and to demonstrate inter-observer agreement. Measurements on maximum intensity projections are not desirable, as larger amounts of fibro-glandular tissue will be perceived as a higher BPE, and thus, BPE will not be measured independently of MRBD. Semi-quantitative measurements of BPE (e.g. percentual enhancement and other metrics based on changes of image intensity) will depend on parenchyma segmentation if performed on the entire breast volume and will depend on the choice of ROI if performed locally.

One limitation of this study is that it is only powered to detect large differences in BPE between the two breast cancer risk groups recruited here. It does not support any significant differences in BPE between the two groups and does not suggest any differences in the perfusion of the normal breast which is associated with the BRCA genes in radiologically normal women. Furthermore, this study has highlighted some of the difficulties in assessing BPE. As expected, there were difficulties in making the measurement operator independent and reproducible. Despite our best efforts, the inter-observer CV was 8.4% for median BPE. However, our high-quality data was acquired with current technology and our methods were optimised for the purpose of BPE calculation. This suggests an intrinsic level of uncertainty in BPE measurements that will hinder its use.

## Conclusion

In conclusion, this carefully designed study did not reveal significant differences or correlations in fibro-glandular tissue volume, MRBD, or median BPE between the two cohorts of radiologically normal women at different levels of breast cancer risk. However, our work supports further investigation to probe the heterogeneity of the parenchymal enhancement. In future work, this non-uniform nature of breast enhancement could be explored in developing alternative quantitative MRI approaches for breast cancer risk stratification.

## Supplementary Information

Below is the link to the electronic supplementary material.

Supplementary file1 (PDF 107 KB)

## Acronyms

2D: Two dimensional
3D: Three dimensional
BPE: Background parenchymal enhancement
FS: Fat-suppressed
MRBD: MRI breast density
XRM: X-ray mammography

## Declarations

### Guarantor

The scientific guarantor of this publication is Dr Maria A Schmidt.

### Statistics and biometry

Catey Bunce and Komel Khabra were consulted as experts in statistics and provided statistical advise. They are authors of this paper. Both experts were affiliated with The Royal Marsden NHS Foundation Trust, Sutton, UK.

### Methodology

• prospective

• observational

• multicentre study
