# Correction: Quantitative background parenchymal enhancement and fibro-glandular density at breast MRI: Association with BRCA status

**DOI:** 10.1007/s00330-023-09819-2

**Published:** 2023-06-26

**Authors:** Rosie Goodburn, Evanthia Kousi, Clarrissa Sanders, Alison Macdonald, Erica Scurr, Catey Bunce, Komel Khabra, Mamatha Reddy, Louise Wilkinson, Elizabeth O’Flynn, Steven Allen, Maria Angélica Schmidt

**Affiliations:** 1 CRUK Cancer Imaging Centre, The Institute of Cancer Research and Royal Marsden Foundation Trust, London, UK; 2 The Royal Marsden NHS Foundation Trust, Sutton, UK; 3 St Georges University Hospitals NHS Foundation Trust, London, UK

**Correction: European Radiology**

**https://doi.org/10.1007/s00330-023-09592-2**

The original version of this article, published on 5 April 2023, unfortunately contained a mistake. In the caption of Fig. 2, the sentence "An enhancement map is shown on the 5th post-contrast image (c), which was the image volume with the highest median pixel value over the segmented parenchyma" was corrected to read "An enhancement map is shown on the 5th post-contrast image (g), which was the image volume with the highest median pixel value over the segmented parenchyma". The original article has been corrected.

