# Supplementary material for: Quantitative background parenchymal enhancement and fibro-glandular density at breast MRI: Association with BRCA status
Source: Eur Radiol. 2023 Apr 5;33(9):6204–12. doi: 10.1007/s00330-023-09592-2 (PMC10415521; doi:10.1007/s00330-023-09592-2)
Supplement: Supplementary file 1 — Supplementary file1 (PDF 107 KB) [file 330_2023_9592_MOESM1_ESM.pdf]

Supplementary Material:

Assessment of Dynamic Range of T1-weighted DCE pulse sequences

The dynamic range of the DCE sequence was investigated in both scanners to with a set of aqueous $CuSO_4$ solutions (T1 range: 30 - 2400 ms) to produce a curve of DCE image intensity against R1 (=1/T1). In order to correctly represent BPE, a linear relationship between the DCE image intensity and R1 must be present over the range of T1 values associated with the breast parenchyma. At 3 T, the unenhanced breast parenchyma is expected to have T1s of the order of 1400 ms [29]. Smooth splines were fitted to the data and curves obtained on both scanners were compared for T1s larger than 100 ms. This range is based on quantitative measurements of T1 on breast lesions [30, 31].

Figure S1 shows the curves produced by the MRI scanners at both sites. The normalised image intensity of the DCE sequences can be considered approximately directly proportional to R_1 for $R_1 < 0.01$ ms^{-1} (or $T_1 > 100$ ms). The curves for the two MRI systems are in agreement: the coefficient of variation of the area under the fitted smoothing spline curves was 0.2%. In addition, the normalised DCE image intensity values for the different T_1 values were compared between the two sites and the coefficient of variation ranged between 0.2% and 5.5%.

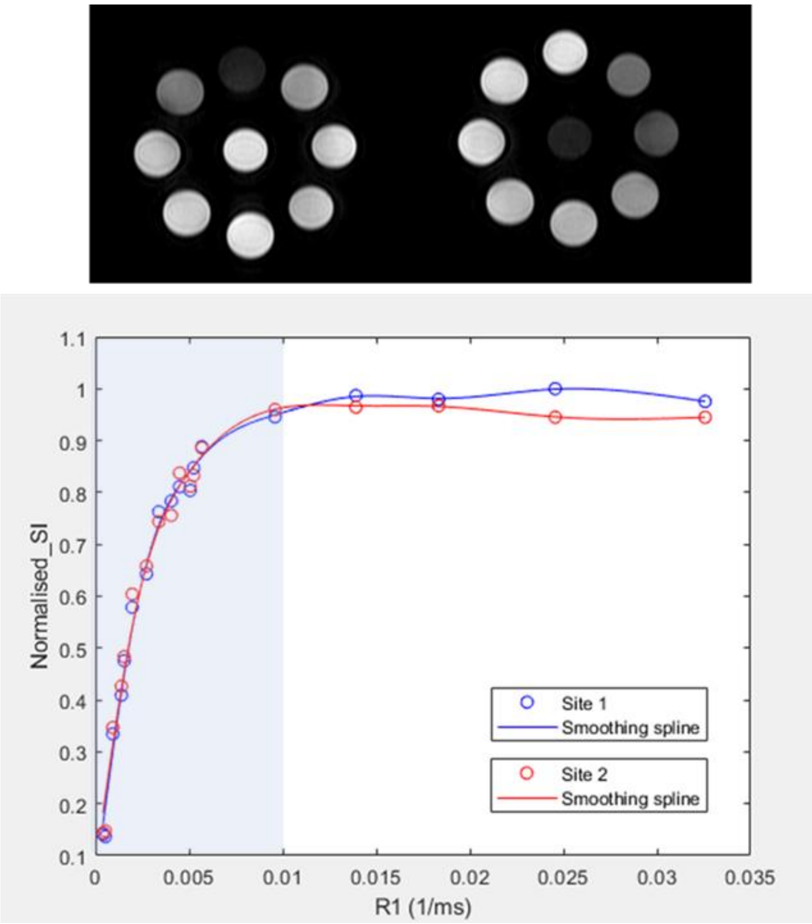

Figure S1: Curves as measured for the two different sites: Normalised DCE image intensity as a function of R1 (ms-1) for test objects with T1 ranging from 30 ms to 2400 ms (upper figure). The fitted smoothing splines (bottom figure) show a good agreement between the two sites. The shaded area shows that the pulse sequences employed exhibit an approximately linear relationship between the image intensity and 1/T1 for the range of T1 values found in breast.

The pulse sequence employed responds to T1 changes for T1 values over 100 ms, and that the relationship between image intensity and 1/T1 is approximately linear. This ensures that the enhancement shown in subtracted images is proportional to contrast uptake and therefore different levels of contrast agent uptake in the patient population can actually be distinguished. In addition, it is also clear that both scanners employed perform comparably and the use of different scanners is unlikely to introduce any bias.

References:

29. Rakow-Penner R, Daniel B, Yu H, et al (2006) Relaxation times of breast tissue at 1.5T and 3T measured using IDEAL. J Magn Reson 23:87–91

30. Leach MO, Boggis CR, Dixon AK, et al (2005) Screening with magnetic resonance imaging and mammography of a UK population at high familial risk of breast cancer: a prospective multicentre cohort study (MARIBS). The Lancet 365:1769–1778
31. Kousi E, Smith J, Ledger AE, et al (2018) Quantitative evaluation of contrast agent uptake in standard fat-suppressed dynamic contrast-enhanced MRI examinations of the breast. Med Phys 45:287–296
